# Transcriptomic and proteomic profiling of Na_V_1.8-expressing mouse nociceptors

**DOI:** 10.3389/fnmol.2022.1002842

**Published:** 2022-10-11

**Authors:** Manuela Schmidt, Julia Regina Sondermann, David Gomez-Varela, Cankut Çubuk, Queensta Millet, Myles J. Lewis, John N. Wood, Jing Zhao

**Affiliations:** ^1^Systems Biology of Pain, Division of Pharmacology and Toxicology, Department of Pharmaceutical Sciences, University of Vienna, Vienna, Austria; ^2^Centre for Experimental Medicine and Rheumatology, William Harvey Research Institute, Barts and The London School of Medicine and Dentistry, Queen Mary University of London, London, United Kingdom; ^3^Centre for Translational Bioinformatics, William Harvey Research Institute, Barts and The London School of Medicine and Dentistry, Queen Mary University of London, London, United Kingdom; ^4^Molecular Nociception Group, Wolfson Institute for Biomedical Research, Division of Medicine, University College London, London, United Kingdom

**Keywords:** Na_V_1.8, nociceptor, dorsal root ganglia (DRG), pain, transcriptome, proteome

## Introduction

Chronic pain is poorly treated in the clinic as most available analgesics have low efficacy and can cause serious side effects. A recent survey on chronic pain shows that one in five European people suffers from chronic pain, thus, the development of new types of analgesic drugs is urgently needed (Alliance, 2017). This depends on a more detailed understanding of the molecular mechanisms underlying pain syndromes. The concept of nociceptors was introduced by neurophysiologist Sir Charles Sherrington in the early twentieth century and their existence was first demonstrated by Edward R. Perl (Sherrington, 1903; Mason, 2007; Wood, 2020). Nociceptors are specialized primary sensory neurons resident in dorsal root ganglia (DRG) and trigeminal ganglia that play a fundamental role in both acute pain and chronic pain conditions (Abrahamsen et al., 2008; Reichling and Levine, 2009; Dubin and Patapoutian, 2010; Middleton et al., 2021). DRG are located outside the blood-brain barrier rendering them an important therapeutic target of chronic pain, in particular for peripherally-acting treatments (Woolf and Ma, 2007; Sapunar et al., 2012; Price et al., 2018). Previous work has established that the expression of the sodium channel Na_V_1.8 defines a distinct subset of nociceptors involved in mechanical and inflammatory pain (Akopian et al., 1999; Wood, 2020).

Many studies have focused on investigating pain-associated gene expression changes in DRG. Microarray profiling and RNA-Seq have been widely employed to investigate the DRG transcriptome in many species such as mouse, rat, primate, and human (Chiu et al., 2014; Gong et al., 2016; Li et al., 2016; Barry et al., 2018; Ray et al., 2018; Megat et al., 2019a,b; Yokoyama et al., 2020; Kupari et al., 2021). Among these reports, some assessed mRNA levels specifically in Na_V_1.8-expressing (Na_V_1.8^+^) nociceptors. For example, Thakur et al. identified 920 transcripts enriched in nociceptors using Na_V_1.8-tdTomato mice combined with magnetic cell sorting (MACS) and RNA-Seq technologies (Thakur et al., 2014). In 2008, we described insights into the nociceptor transcriptome of Na_V_1.8Cre^+/−^; ROSA26-flox-stop-flox-DTA (Diphtheria toxin fragment A) mutant mice (DTA), in which Na_V_1.8^+^ DRG neurons, representing mainly nociceptors, were specifically ablated by expression of DTA. More recently, subtypes of DRG neurons, including nociceptors, were identified, and distinguished by comprehensive single-cell RNA-Seq profiles in mouse and primates generating a highly valuable reference atlas of gene expression in DRG and beyond (Usoskin et al., 2015; Zeisel et al., 2018; Kupari et al., 2021). Furthermore, Megat et al. reported the translatome of Na_V_1.8^+^ DRG neurons using translating ribosome affinity purification (TRAP) and compared their data to the transcriptome elucidated by RNA-Seq (Megat et al., 2019a). Importantly, they observed only minor correlations between the translatome and transcriptome. It is well-known that transcript levels only show limited correspondence with protein abundance given diverse cellular buffering mechanisms, e.g., regulation at the level of transcription, translation, post-translation, and degradation or protein stability (Liu et al., 2016; Reimegård et al., 2021). In functional terms, proteins are the building blocks of a cell, and they are crucially implicated in determining phenotypes, including pain-related outcomes and behaviors. However, compared to the transcriptome of nociceptors, the Na_V_1.8^+^ nociceptor proteome remains undefined.

**Figure 1:**
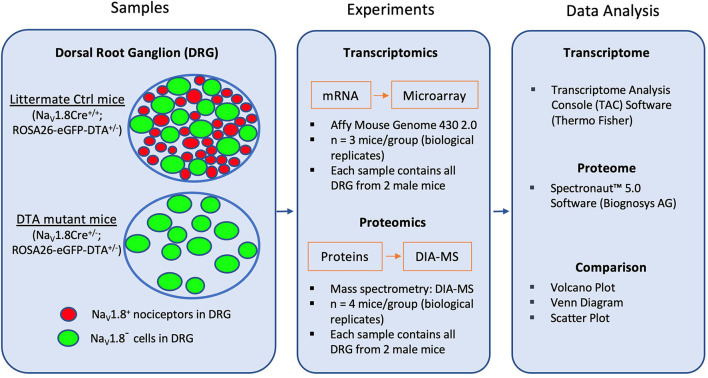
Schematic diagram for transcriptome and proteome analysis in Na_V_1.8-expressing (Na_V_1.8^+^) DRG neurons (mainly nociceptors). The diagram shows the key steps of the workflow. First, RNA and protein samples from DRG were collected from both DTA mutant (Na_V_1.8Cre^+/−^; ROSA26-eGFP-DTA^+/−^) mice, in which Na_V_1.8^+^ nociceptors have been ablated, and littermate control (Ctrl) mice (Nav1.8Cre^+/+^; ROSA26-eGFP-DTA^+/−^). Isolated RNA and proteins were identified with Affymetrix microarray and Data Independent Acquisition-Mass Spectrometry (DIA-MS), respectively. Generated datasets were analyzed using Transcriptome Analysis Console (TAC) software for transcriptome profiling, and Spectronaut™ Software (Biognosys AG) for proteome profiling.

We have recently shown the immense potential of defining pain-associated proteome dynamics in DRG by data-independent acquisition mass spectrometry (DIA-MS) (Rouwette et al., 2016; Barry et al., 2018; De Clauser et al., 2022). Here, we employed DIA-MS to reveal the previously unknown protein set-up of Na_V_1.8^+^ nociceptors by using aforementioned DTA mice (Abrahamsen et al., 2008). Following the workflow chart shown in Figure 1, we, in parallel, re-analyzed the raw microarray data (ArrayExpress: E-MEXP-1622) obtained in our previous study (Abrahamsen et al., 2008) using the latest version of Transcriptome Analysis Console (TAC) Software 4.0.2. Furthermore, the two datasets were compared and are presented here with Volcano plots, Venn diagrams, Pearson scatter plots and bar charts. The top 50 nociceptor-enriched transcripts/proteins can be found in Supplementary Tables 1, 2. Overall, this study provides a valuable resource atlas covering transcripts and proteins in Na_V_1.8^+^ nociceptors of mouse DRG.

## Materials and methods

### Animals

To ablate the neuronal population of DRG neurons expressing Na_V_1.8, heterozygous Na_V_1.8Cre knock-in mice (JAX stock #036564) (Nassar et al., 2004; Stirling et al., 2005) were crossed to homozygous ROSA26-eGFP-DTA mice (JAX stock #006331), in which an enhanced green fluorescent protein (eGFP) and a loxP-flanked STOP cassette following a diphtheria toxin fragment A were inserted in the ROSA26 locus (Ivanova et al., 2005; Abrahamsen et al., 2008). Upon exposure to Cre recombinase in Na_V_1.8Cre-expressing DGR neurons, the floxed STOP fragment was removed resulting in an ablation of Na_V_1.8-expressing cells in DTA mice. In contrast, Cre-negative littermates were employed as control mice: Na_V_1.8Cre^+/−^; ROSA26-eGFP-DTA^+/−^ mutant mice (DTA) represent the “nociceptor-deficient group” and Na_V_1.8Cre^+/+^; ROSA26-eGFP-DTA^+/−^ were used as littermate control mice (Ctrl), respectively (Figure 1). Genotyping was performed as previously described (Abrahamsen et al., 2008).

### RNA Microarray

mRNA Microarray experiments were performed as described in our previous study (Abrahamsen et al., 2008). Raw data were stored in ArrayExpress (E-MEXP-1622) and Transcriptome Analysis Console (TAC) Software (version 4.0.2) was used for extracting the raw data. The RMA (Robust Multi-array Average) method (Irizarry et al., 2003) was applied for background correction and normalization of probe values. The non-normalized and normalized probe values for each sample for each probe in the microarray datasets can be found in Supplementary Tables 3, 4, respectively. Cluster analysis of transcriptome data shows that the samples fall into two clear groups (Supplementary Figure 1) Prior to differential expression analysis, the distribution of the coefficient of variation (CV) for genes with multiple probes was investigated. The result shows that the variability between the probes representing the same gene was low (CV values between −0.02 and 0.05) (Supplementary Figure 2). Therefore, we assigned the highest fold-change of probes to their corresponding genes. A list of all identified transcripts can be found in Supplementary Table 5.

### Sample preparation for DIA-MS

DRG were isolated from eight DTA males, aged between 8- and 12-week-old, and eight littermate male controls, i.e., four biological replicates with two mice/replicate in total. Protein samples of DRG were prepared as described previously (Barry et al., 2018). All reagents were obtained from Roth. All DRG samples were stored at −80° until further use.

### DIA-MS and data analysis

All reagents were purchased from Sigma-Aldrich if not stated otherwise. All steps of DIA-MS and its analysis were performed by Biognosys AG (Zuerich, Switzerland) essentially as described (Bruderer et al., 2015; Rouwette et al., 2016) with the following modifications: For Global HRM profiling, 2 μg of peptides were injected *via* an in-house packed C18 column (Dr. Maisch ReproSil Pur, 1.9 μm particle size, 120 Å pore size; 75 μm inner diameter, 50 cm length, New Objective) on a Thermo Scientific Easy nLC 1200 nano-liquid chromatography system connected to a Thermo Scientific Fusion Lumos Tribrid mass spectrometer equipped with a standard nano-electrospray source. LC solvents were A: 1% acetonitrile in water with 0.1% FA; B: 15% water in acetonitrile with 0.1% FA. The non-linear LC gradient was 1–55% solvent B in 120 min followed by 55–90% B in 10 s, 90% B for 10 min, 90–1% B in 10 s and 1% B for 5 min. A DIA method with one full range survey scan and 40 DIA windows was adopted from a previous study (Bruderer et al., 2015). HRM mass spectrometric data were analyzed using Spectronaut software (Biognosys). The false discovery rate on peptide and protein level was set to 1%, data was filtered using row-based extraction. Data quality was analyzed with directDIA^TM^ analysis using Spectronaut X with default normalization settings (Supplementary Figure 3) indicating high acquisition quality. Thus, only minor normalization was carried out (Supplementary Figure 3A). Furthermore, unsupervised clustering showed a clear separation of samples into two groups, i.e., littermate controls (Ctrl) and DTA mice, as expected (Supplementary Figure 3B). Data analysis was performed using mouse UniProt fasta downloaded 2018-07-01. We used two analysis pipelines as described previously (De Clauser et al., 2022): a spectral library-based search and the directDIA^TM^ workflow developed at Biognosys using Biognosys Factory settings in Spectronaut X. Label-free quantitation was executed on MS2-level using the area under the curve and data were filtered by *Q*-value sparse (precursors robustly found in at least one sample). Statistical testing of differential protein abundances between conditions was calculated in Spectronaut X for each protein ID by performing a pairwise *t*-test. Benjamini-Hochberg (BH)-adjusted *P*-values (*Q*-values) were used for multiple testing and significantly altered proteins were defined by setting a cut-off of *Q* < 0.05. Significantly regulated proteins from both analysis pipelines were pooled in a combined candidate list and duplicates as well as single-peptide-hits were removed. Any potential keratin and serum albumin contaminations were also removed. A list of all quantified proteins and raw data using both analysis pipelines can be found in Supplementary Table 6.

### Data analysis and statistics

Raw data from mRNA Microarray and protein DIA-MS were analyzed with TAC software (4.0.2) and Spectronaut X software, respectively. Further data analysis was performed with GraphPad Prism nine to generate volcano plots, Venn diagrams, bar graphs, and scatter plots for comparative data analysis. All symbols of transcripts and proteins were converted to Mouse Genome Informatics (MGI) approved gene symbols (http://www.informatics.jax.org/batch) for ease of comparison among the two datasets.

All values are presented as Mean ± S.E.M. Data were analyzed by two-way ANOVA and Student's *t-*test. Differences were considered significant at False Discovery Rate (FDR) adjusted *P* < 0.05 for transcriptome data. For proteome analysis, Benjamini-Hochberg (BH)-adjusted *P*-values (*Q*-values) were used for multiple testing and significantly altered proteins were defined by setting a cut-off of *Q* < 0.05 as previously described (De Clauser et al., 2022).

## Transcriptomic profiling of DRG

**Figure 2:**
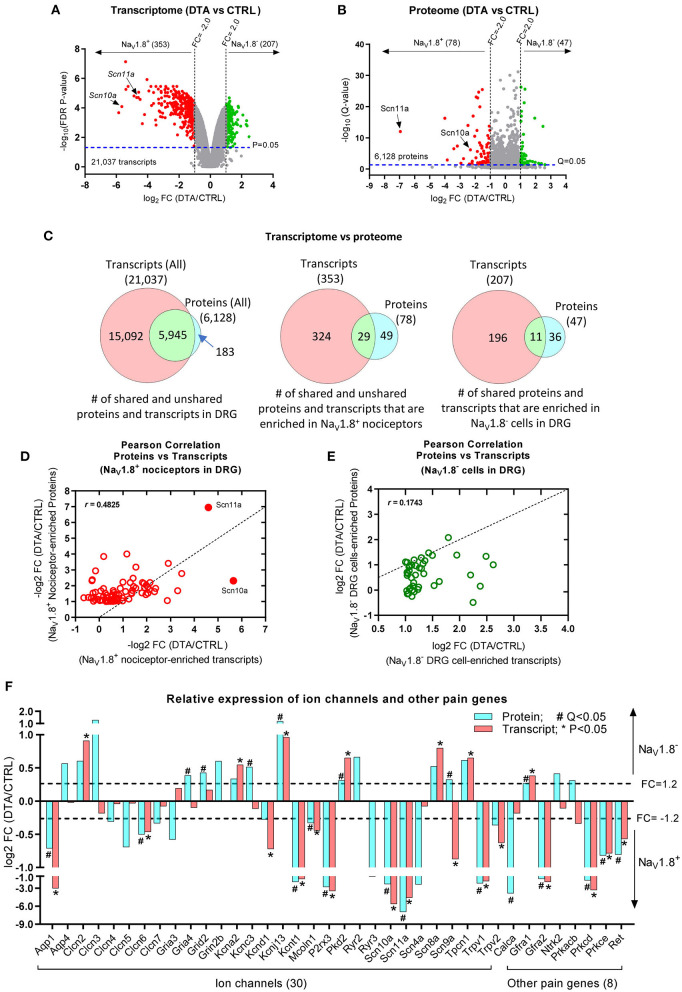
Comparison of the transcriptome and proteome. **(A)** Volcano plot shows the average fold-change (log_2_FC) in expression levels of transcripts *vs*. FDR *P*-values (log_10_FDR *P*-value). Results are filtered by FC: FC < −2.0, red dots; FC > 2.0, green dots; all with FDR *P-*value < 0.05 as indicated by the dashed line. **(B)** Volcano plot shows the average log_2_FC in relative protein abundance *vs. Q*-values (log_10_; *P*-values corrected for multiple-testing). Results are filtered by FC: FC < −2.0, red dots; FC > 2.0, green dots; all with *Q-*value < 0.05 as indicated by the dashed line. **(C)** Venn diagrams indicate the numbers of transcripts and proteins quantified in DRG (left panel), enriched in Na_V_1.8^+^ nociceptors (middle panel), and enriched in Na_V_1.8^−^ DRG cells (right panel), non-nociceptors. **(D,E)** Scatter Plots (Pearson correlation) show the correlation between proteome and transcriptome data: moderate correlation (*r* = 0.4825) in Na_V_1.8^+^ nociceptors (D) and low correlation (*r* = 0.1743) in Na_V_1.8^−^ cells of DRG (E). **(F)** Bar graphs show the comparison of protein *vs*. transcript levels (log_2_FC) of a selection of ion channels and other pain genes being either enriched in Na_V_1.8^+^ nociceptors or in Na_V_1.8^−^ DRG cells (DTA *vs*. Ctrl). *FDR *P*-value < 0.05 in the transcriptome dataset; #*Q*-value < 0.05 in the proteome dataset.

To study the gene expression at mRNA level in DRG, especially in Na_V_1.8^+^ nociceptors (Stirling et al., 2005), we re-analyzed our previous raw DNA microarray data (Abrahamsen et al., 2008) using the latest version (4.0.2) of Transcriptome Analysis Console (TAC) software (Thermo Fisher Scientific). In total, 21,037 transcripts were identified in DRG, among which 353 (1.68% of total transcripts) were significantly down-regulated (Fold-Change, FC < −2.0; FDR adjusted *P-*value < 0.05) in DTA mice (Figure 2A), suggesting that these transcripts were enriched in Na_V_1.8^+^ nociceptors in DRG. As expected, both nociceptive sodium channels Na_V_1.8 (*Scn10a*) and Na_V_1.9 (*Scn11a*) were highly enriched in nociceptors (Figure 2A; Supplementary Table 1). In contrast, 207 transcripts (0.98% of total transcripts) were up-regulated (FC > 2.0; FDR adjusted *P-*value < 0.05) in DRG of DTA mice, indicating that those transcripts exhibit very low to no expression in Na_V_1.8^+^ neurons. Rather, these are likely primarily expressed in Na_V_1.8-negative (Na_V_1.8^−^) cells, including large-diameter DRG neurons and satellite glial cells of DRG. The top 50 transcripts, which are enriched in Na_V_1.8^+^ DRG neurons are listed in Supplementary Table 1. All identified transcripts can be found in Supplementary Table 5.

## Proteomic profiling of DRG

Next, we deeply profiled the proteome of DRG neurons using our established DIA-MS workflow described previously (Barry et al., 2018). As expected, unsupervised clustering showed a clear separation of samples into two groups, i.e., littermate controls (Ctrl) and DTA mice (Supplementary Figure 3B). Overall, we quantified 6,128 proteins (Supplementary Table 6) among which 78 (0.13% of total proteins) were significantly down-regulated (FC < −2.0; *Q-*value < 0.05) in DTA mice (Figure 2B), indicating that these 78 proteins (Supplementary Table 7) were enriched in Na_V_1.8^+^ nociceptors. In line with our transcriptome analysis, Na_V_1.8 (*Scn10a*) and Na_V_1.9 (*Scn11a*) appeared to be highly enriched in nociceptors. In contrast, 47 proteins (Supplementary Table 8) (0.77% of total proteins) were significantly up-regulated (FC > 2.0; *Q-*value < 0.05) in DRG of DTA mice, suggesting that these proteins are primarily expressed in Na_V_1.8^−^ cells in DRG (likely large DRG neurons and/or satellite cells). The top 50 proteins, which were enriched in Na_V_1.8^+^ DRG neurons, are listed in Supplementary Table 2. All quantified proteins and raw datasets can be found in Supplementary Table 6.

However, despite successful quantitation of diverse membrane proteins, our here presented proteome dataset is not complete. Although we provide fingerprints for many pain-related ion channels and receptors (e.g., sodium channels Na_V_1.8 and Na_V_1.9; Trpv1; NGF receptor), our data lacks information on several others such as Trpa1, Trpm8, Piezo2 known to be expressed in DRG. The reasons can be manifold, ranging from relative low expression levels to insufficient solubilization or localization in detergent-resistant microdomains—all factors known to render membrane protein analysis challenging (Lu et al., 2009; Aebersold and Mann, 2016; Liu et al., 2018). Nonetheless, and compared with published proteomics studies on DRG (including our previous ones) (Rouwette et al., 2016; Barry et al., 2018), our dataset constitutes a unique and thus far most comprehensive proteome resource of Na_V_1.8^+^ nociceptors for the pain community. Moreover, the nature of DIA-MS profiling allows any experimenter to employ our data for *in-silico* data analysis without requiring new experiments (Bruderer et al., 2017). Therefore, the depth of protein profiling will steadily improve once experiments in DIA-mode are commonly used by the pain community.

## Comparison of transcripome and proteome datasets

We then directly compared our Na_V_1.8^+^ nociceptor proteome and transcriptome datasets. The Venn diagram (left panel in Figure 2C) shows that 97% (5,945 out of 6,128) proteins could be matched to their corresponding transcripts. The remaining unmatched 183 proteins are listed in Supplementary Table 9. Of note, 127 of these unmatched proteins can be found in the RNA-seq dataset generated by Usoskin et al. (2015), suggesting that these candidates are indeed expressed in DRG but were not included in our original microarray assay (Abrahamsen et al., 2008). As expected, the transcriptome dataset was much more comprehensive than the protein dataset owing to technical reasons mentioned above. Consequently, we did not obtain protein abundance data on 2/3 of detected transcripts, including pain-relevant genes such as TrpA1 and Trpm8. The middle panel in Figure 2C illustrates that only 29 out of 78 (37.2%) proteins (Supplementary Table 7) enriched in Na_V_1.8^+^ nociceptors appeared to be nociceptor-enriched on the mRNA level. Similarly, the overlap of proteins and transcripts enriched in Na_V_1.8^−^ DRG cells was relatively low: only 11 out of 47 (23.4%) proteins (Supplementary Table 8) could be matched on the transcript level (right panel in Figure 2C). To directly assess the correlation between our transcript and protein datasets, we calculated Pearson's correlation coefficients. The two datasets exhibited moderate correlation (*r* = 0.4825) in Na_V_1.8^+^ nociceptors (Figure 2D), and low correlation (*r* = 0.1743) in Na_V_1.8^−^ cells of DRG (Figure 2E). This low correlation of transcriptome and proteome is a well-known fact given the existence of cellular buffering mechanisms on all levels—transcriptional, translational, and post-translational and considering protein degradation/stability (Li et al., 2016; Reimegård et al., 2021). As expected, a comparison with published datasets on the transcriptome (Usoskin et al., 2015) and the translatome of Na_V_1.8^+^ nociceptors (Megat et al., 2019a) showed differing overlap with both our transcriptomic dataset (16.1%) and proteomic dataset (15.2%), respectively (Supplementary Table 10): While transcriptome data were highly similar (overlap 85%), this was not the case for transcriptome *vs*. translatome comparisons (overlap 2.1%). Moreover, our Na_V_1.8^+^ nociceptor proteome data only shared few candidates with the transcriptome (11.5%) and the translatome (2.1%) (Supplementary Table 10). Taken together, these results strongly advocate for the integration of multi-omics approaches to obtain a detailed understanding of the molecular set-up of tissues and distinct cell types relevant for pain.

In a next step, we compared transcript and protein levels of a selection of ion channels and representative pain genes. For the most part transcript and protein data were well-correlated in respect to their enrichment in Na_V_1.8^+^ nociceptors and Na_V_1.8^−^ DRG cells, respectively. For example, Na_V_1.8 (*Scn10a*), Na_V_1.9 (*Scn11a*), *Trpv1, P2rx3*, PKC Delta (*Prkcd*) and PKC Epsilon (*Prkce*), c-RET (*RET*), GDNF Receptor A2 (*Gfra2*), and Aquaporin 1 (*Aqp1*) were significantly enriched in Na_V_1.8^+^ nociceptors (Figure 2F), while potassium channels Kcna2 and Kcnj13, Polycystin-2 (*Pkd2*), *Scn8a*, and GDNF Receptor A1 (*Gfra1*) were enriched in Na_V_1.8^−^ cells of DRG. In contrast, some molecules showed opposite trends of transcript and protein levels, such as Na_V_1.7 (*Scn9a*) and PKA Beta (*Prkacb*), a finding that awaits further investigation by other methods. We further assessed the expression of these 29 candidates, which are shared among our transcriptome and proteome datasets (Supplementary Table 11), by comparison to a single cell RNA-Seq database (www.mousebrain.org) (Zeisel et al., 2018). This comparison confirmed here shown enrichment in nociceptors (Supplementary Table 11) for most candidates and also highlights some candidates, which have previously not been shown to exhibit an expression pattern typical for Na_V_1.8^+^ nociceptors, such as Diacylglycerol kinase gamma (*Dgkg*) or Regulator of G-protein signaling 3 (*Rgs3*). Future immunostainings with validated specific antibodies are required to decipher their expression pattern in detail.

## Conclusion

This comparative profiling study at both mRNA and protein levels in mouse Na_V_1.8^+^ nociceptors provides a unique and highly valuable source for further investigations on the molecular basis of somatosensation and pain. Nonetheless, this resource is not complete and needs to be integrated with future multi-omics efforts of the scientific community to obtain a full understanding of the molecular set-up of Na_V_1.8^+^ nociceptors.

## Data availability statement

All proteomics raw data and sample report files have been deposited to the ProteomeXchange Consortium *via* the PRIDE partner repository (Perez-Riverol et al., 2019) with the dataset identifier PXD034447.

## Ethics statement

All animal procedures were performed by licensed individuals and conformed to UK Home Office regulations in accordance with the Animals (Scientific Procedures) Act 1986. The project was overseen by the UCL Ethics Committee.

## Author contributions

MS, JW, and JZ were involved in conceptualization, formal analysis, investigation, methodology, and writing the manuscript. DG-V, JS, and JZ were involved in investigation and data collection. CÇ, ML, MS, and JZ were involved in data analysis. QM were involved in investigation. All authors contributed to data discussion and read and approved the manuscript.

## Funding

This work was funded by a Wellcome Collaborative Award (200183/Z/15/Z) and Research Award—Arthritis Research UK [21950]. This work was also funded by the Max Planck Society and the University of Vienna. CÇ was supported by an MRC grant award (MR/V012509/1).

## Conflict of interest

The authors declare that the research was conducted in the absence of any commercial or financial relationships that could be construed as a potential conflict of interest.

## Publisher's note

All claims expressed in this article are solely those of the authors and do not necessarily represent those of their affiliated organizations, or those of the publisher, the editors and the reviewers. Any product that may be evaluated in this article, or claim that may be made by its manufacturer, is not guaranteed or endorsed by the publisher.
