# Supplementary material for: Transcriptomic and proteomic profiling of NaV1.8-expressing mouse nociceptors
Source: Front Mol Neurosci. 2022 Oct 11;15:1002842. doi: 10.3389/fnmol.2022.1002842 (PMC9593034; doi:10.3389/fnmol.2022.1002842)
Supplement: Supplementary file 11 [file Table_11.pdf]

Supplementary Table 11: 29 Transcripts/Proteins enriched in $Na_V1.8$-expressing DRG Neuronss

Transcriptome vs Proteome

		Transcripts		Proteins	
No	Symbol	Fold-Change (DTA/Ctrl)	FDR P-Val	Fold-Change (DTA/Ctrl)	Q-value
1	Acpp	-7.518104	0.000037	-10.653613	0
2	Arhgap28	-4.390761	0.000004	-4.594784	0.004716
3	Camk2a	-2.238842	0.000171	-16.040381	0
4	Clgn	-2.155886	0.000442	-3.10821	0
5	Dgkg	-5.017199	0.000021	-3.081118	0.000018
6	Dgkh	-4.136786	0.000022	-3.822114	0
7	Dgki	-2.684322	0.000042	-5.568794	0
8	Dgkz	-3.750441	0.000018	-3.69622	0
9	Eml1	-5.175388	0.000004	-3.56907	0
10	Fxyd2	-2.583285	0.000034	-2.937812	0
11	Gfra2	-3.904673	0.000016	-2.711791	0.000002
12	Kcnt1	-2.720936	0.000047	-3.865256	0.000266
13	Nedd4l	-3.935441	0.000011	-4.087266	0
14	Osbpl3	-4.304144	0.000129	-3.400133	0
15	P2rx3	-11.08769	0.000004	-6.823287	0.000479
16	Phf24	-3.669058	0.00003	-4.387869	0
17	Pirt	-2.90374	0.000019	-3.61859	0.000003
18	Plcb3	-4.139667	0.000009	-2.890539	0
19	Plcxd3	-2.570047	0.00003	-2.841155	0.004504
20	Pqlc3	-2.368454	0.000021	-2.247864	0.000009
21	Prkcd	-9.890721	0.000026	-3.215095	0
22	Prkcq	-7.28081	0.000093	-2.083968	0.000023
23	Ptrh1	-2.479172	0.000095	-4.588019	0.029506
24	Rgs10	-3.480715	0.000021	-2.926472	0
25	Rgs3	-2.911075	0.00002	-9.058443	0
26	Scg3	-2.636118	0.000022	-3.268107	0
27	Scn10a	-50.14744	0.000082	-4.983505	0.000001
28	Scn11a	-24.20722	0.00002	-123.96514	0
29	Trpv1	-3.522796	0.000262	-4.578864	0.003117

Single-cell RNA-Seq (1-10)

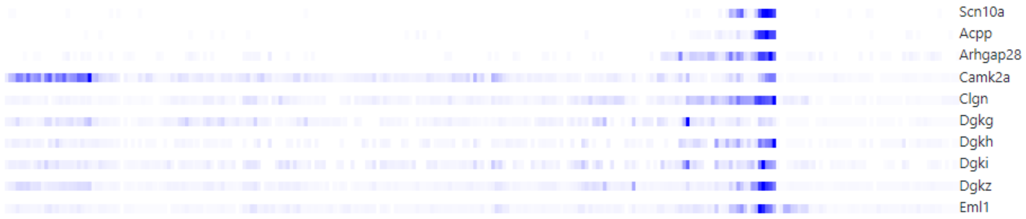

Index	Name	Description	Scn10a	Acpp	Arhgap28	Camk2a	Clgn	Dgkg	Dgkh	Dgki	Dgkz	Eml1
198	PSPEP8	Peptidergic (TrpM8), DRG	0.00	0.142	0.179	0.109	0.501	0.0369	0.321	0.106	0.929	1.32
199	PSPEP7	Peptidergic (TrpM8), DRG	0.0788	0.282	0.0937	0.0778	0.360	0.111	0.0623	0.0310	0.501	0.0932
200	PSPEP6	Peptidergic (TrpM8), DRG	0.00	0.0933	0.130	0.261	0.463	0.111	0.0738	0.0367	0.539	0.204
201	PSPEP5	Peptidergic (PEP1.2), DRG	0.199	0.0324	0.401	0.666	0.601	0.235	0.233	0.0330	0.567	0.335
202	PSPEP2	Peptidergic (PEP1.3), DRG	1.56	0.148	0.854	1.26	1.05	0.303	0.0979	0.0873	1.90	1.44
203	PSPEP4	Peptidergic (PEP1.1), DRG	1.16	0.0162	0.325	0.599	0.744	0.274	0.145	0.00857	0.786	1.36
204	PSPEP3	Peptidergic (PEP1..4), DRG	2.51	0.556	0.729	2.08	1.51	0.762	0.293	0.0539	4.48	2.16
205	PSPEP1	Peptidergicv (PEP2), DRG	3.28	0.116	0.527	0.911	1.09	0.269	0.102	0.0381	2.67	2.82
206	PSNF3	Neurofilament (NF2/3), DRG	0.613	0.0973	0.194	0.0311	1.03	0.129	0.0320	0.0320	1.16	1.35
207	PSNF2	Neurofilament (NF4/5), DRG	0.0350	0.0183	0.123	0.0545	1.02	0.140	0.123	0.0696	0.929	0.157
208	PSNF1	Neurofilament (NF1), DRG	0.0263	0.501	0.369	0.160	0.947	0.00	0.0263	0.104	5.26	1.05
209	PSNP1	Non-peptidergic (TH), DRG	1.08	2.91	0.561	0.132	2.00	<=0.00264	0.0850	0.0210	5.06	2.04
210	PSNP2	Non-peptidergic (NP1.1), DRG	3.71	4.16	1.22	1.67	2.25	0.946	0.472	0.261	10.8	7.28
211	PSNP3	Non-peptidergic (NP1.2), DRG	5.28	4.24	1.23	2.49	2.02	0.701	0.344	0.510	13.8	6.27
212	PSNP4	Non-peptidergic (NP2.1), DRG	5.47	5.07	1.07	4.25	1.87	0.490	0.444	0.220	10.8	4.16
213	PSNP5	Non-peptidergic (NP2.2), DRG	4.99	3.66	1.65	5.04	1.74	0.717	0.477	0.203	9.58	3.71
214	PSNP6	Non-peptidergic (NP3), DRG	4.00	3.59	0.918	4.89	2.91	0.295	0.900	0.165	6.97	1.71

Data was extracted from MouseBrain.org

Supplementary Table 11: 29 Transcripts/Proteins enriched in $Na_V1.8$-expressing DRG Neurons

Single-cell RNA-Seq (11-20)

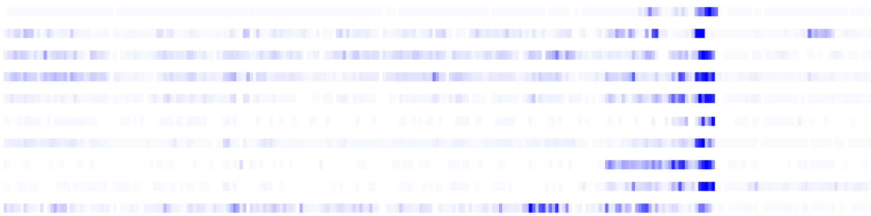

Index	Name	Description	Fxyd2	Gfra2	Kcnt1	Nedd4l	Osbpl3	P2rx3	Phf24	Pirt	Plcb3	Plcxd3
198	PSPEP8	Peptidergic (TrpM8), DRG	0.618	0.179	0.00	0.178	1.03	0.00	2.57	6.75	0.179	0.107
199	PSPEP7	Peptidergic (TrpM8), DRG	0.333	0.281	0.00	0.141	0.359	0.00	1.05	2.09	0.0624	0.266
200	PSPEP6	Peptidergic (TrpM8), DRG	0.428	0.148	0.00	0.278	0.500	0.0368	0.737	3.44	0.0738	0.0930
201	PSPEP5	Peptidergic (PEP1.2), DRG	6.18	0.00	0.200	0.333	2.00	0.00	0.737	5.80	0.233	0.167
202	PSPEP2	Peptidergic (PEP1.3), DRG	14.2	0.0302	0.372	1.20	3.42	0.0881	1.95	14.0	0.716	0.314
203	PSPEP4	Peptidergic (PEP1.1), DRG	4.85	0.0162	0.333	0.375	1.95	0.0595	1.39	7.32	0.453	0.291
204	PSPEP3	Peptidergic (PEP1..4), DRG	15.7	0.0650	0.553	2.81	3.17	0.130	2.59	12.6	1.75	0.0870
205	PSPEP1	Peptidergicv (PEP2), DRG	5.85	0.0650	0.359	2.47	3.64	0.141	3.64	13.7	0.616	0.0260
206	PSNF3	Neurofilament (NF2/3), DRG	0.808	0.258	0.579	0.774	0.806	0.290	2.61	5.94	0.193	0.0320
207	PSNF2	Neurofilament (NF4/5), DRG	0.523	0.123	0.105	0.438	0.193	0.00	1.49	2.81	0.176	0.0880
208	PSNF1	Neurofilament (NF1), DRG	34.4	0.921	0.367	0.158	0.920	0.00	2.31	2.11	0.395	0.0260
209	PSNP1	Non-peptidergic (TH), DRG	60.0	5.68	0.411	3.48	1.74	0.0813	15.8	3.53	0.0426	0.415
210	PSNP2	Non-peptidergic (NP1.1), DRG	55.1	5.80	1.97	2.83	5.61	0.305	18.9	10.6	2.18	1.28
211	PSNP3	Non-peptidergic (NP1.2), DRG	98.2	5.80	2.41	3.58	3.61	0.965	21.4	14.3	2.46	1.34
212	PSNP4	Non-peptidergic (NP2.1), DRG	119	0.0441	2.20	3.91	5.29	0.244	4.31	16.4	2.60	0.756
213	PSNP5	Non-peptidergic (NP2.2), DRG	80.8	0.0232	1.84	2.48	5.12	0.556	10.3	14.5	2.02	0.455
214	PSNP6	Non-peptidergic (NP3), DRG	65.3	0.0673	1.03	3.52	4.72	1.21	3.82	7.19	2.59	0.0610

Single-cell RNA-Seq (21-29)

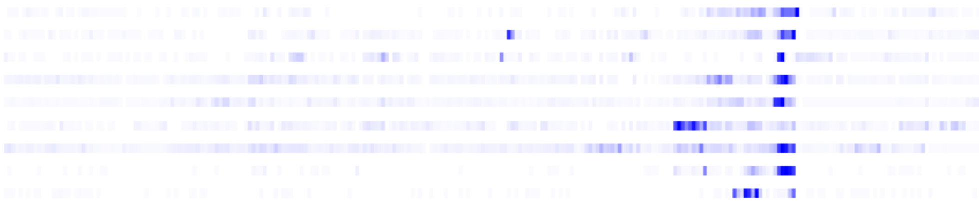

Index	Name	Description	Pqlc3	Prkcd	Prkcq	Ptrh1	Rgs10	Rgs3	Scg3	Scn11a	Trpv1
198	PSPEP8	Peptidergic (TrpM8), DRG	0.501	0.499	0.00	0.0712	10.2	0.0710	1.03	0.00	2.21
199	PSPEP7	Peptidergic (TrpM8), DRG	0.297	0.139	0.00	0.0472	13.1	0.109	0.296	0.00	0.672
200	PSPEP6	Peptidergic (TrpM8), DRG	0.390	0.333	0.0183	0.0927	12.6	0.111	0.261	0.0552	0.314
201	PSPEP5	Peptidergic (PEP1.2), DRG	0.367	0.801	0.00	0.0671	2.78	0.133	1.00	0.434	3.07
202	PSPEP2	Peptidergic (PEP1.3), DRG	0.745	1.74	0.00	0.196	5.50	0.499	1.25	0.852	2.68
203	PSPEP4	Peptidergic (PEP1.1), DRG	0.582	1.71	0.00	0.120	2.60	0.487	0.829	1.55	1.02
204	PSPEP3	Peptidergic (PEP1..4), DRG	0.935	1.97	0.0211	0.120	5.21	0.337	1.06	0.901	3.26
205	PSPEP1	Peptidergicv (PEP2), DRG	0.757	1.55	0.102	0.116	7.26	0.320	1.50	0.513	0.563
206	PSNF3	Neurofilament (NF2/3), DRG	0.0966	0.0658	0.00	0.00	6.11	0.0320	1.68	0.290	0.00
207	PSNF2	Neurofilament (NF4/5), DRG	0.105	0.0696	0.00	0.0530	3.01	0.0702	1.61	0.122	0.0522
208	PSNF1	Neurofilament (NF1), DRG	0.447	0.104	0.00	0.238	3.65	0.131	2.50	0.0256	0.00
209	PSNP1	Non-peptidergic (TH), DRG	0.560	0.665	0.0140	0.774	57.9	0.114	3.15	0.667	0.0143
210	PSNP2	Non-peptidergic (NP1.1), DRG	1.60	2.56	2.77	1.43	61.1	0.222	9.77	3.39	0.0561
211	PSNP3	Non-peptidergic (NP1.2), DRG	1.40	7.02	3.51	1.79	72.4	0.318	12.6	4.80	0.0522
212	PSNP4	Non-peptidergic (NP2.1), DRG	1.42	5.09	0.0225	2.07	18.3	0.222	11.3	4.93	0.0443
213	PSNP5	Non-peptidergic (NP2.2), DRG	1.36	4.97	0.00	0.933	20.2	0.114	7.14	4.57	0.728
214	PSNP6	Non-peptidergic (NP3), DRG	2.57	9.67	0.0154	0.539	11.1	0.469	9.43	3.77	1.95

Data was extract from MouseBrain.org
