# Supplementary material for: Transcriptomic and proteomic profiling of NaV1.8-expressing mouse nociceptors
Source: Front Mol Neurosci. 2022 Oct 11;15:1002842. doi: 10.3389/fnmol.2022.1002842 (PMC9593034; doi:10.3389/fnmol.2022.1002842)
Supplement: Supplementary file 12 [file Image_1.pdf]

Supplementary Figure 1

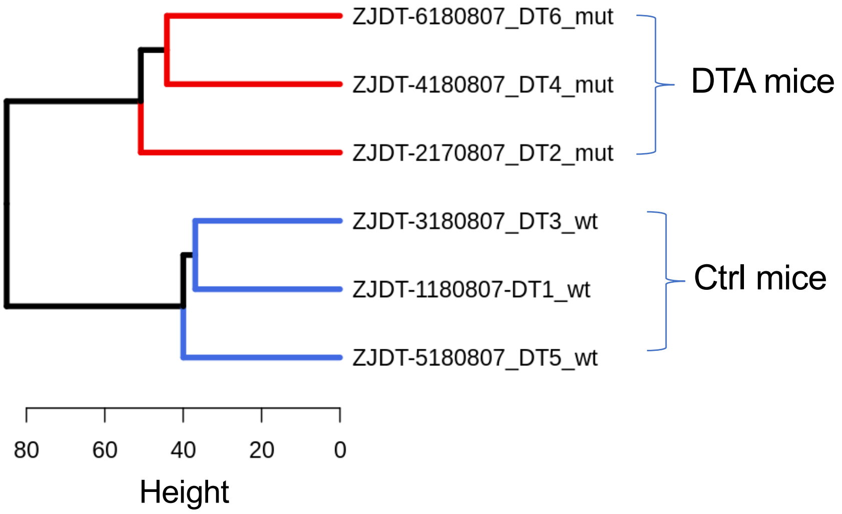

Supplementary Figure 1. Cluster analysis of transcriptome data. Hierarchical clustering shows that samples fall into two clear groups, i.e. DTA mutant mice (in red) and littermate control mice (in blue).
