# Supplementary material for: Transcriptomic and proteomic profiling of NaV1.8-expressing mouse nociceptors
Source: Front Mol Neurosci. 2022 Oct 11;15:1002842. doi: 10.3389/fnmol.2022.1002842 (PMC9593034; doi:10.3389/fnmol.2022.1002842)
Supplement: Supplementary file 13 [file Image_2.pdf]

Supplementary Figure 2

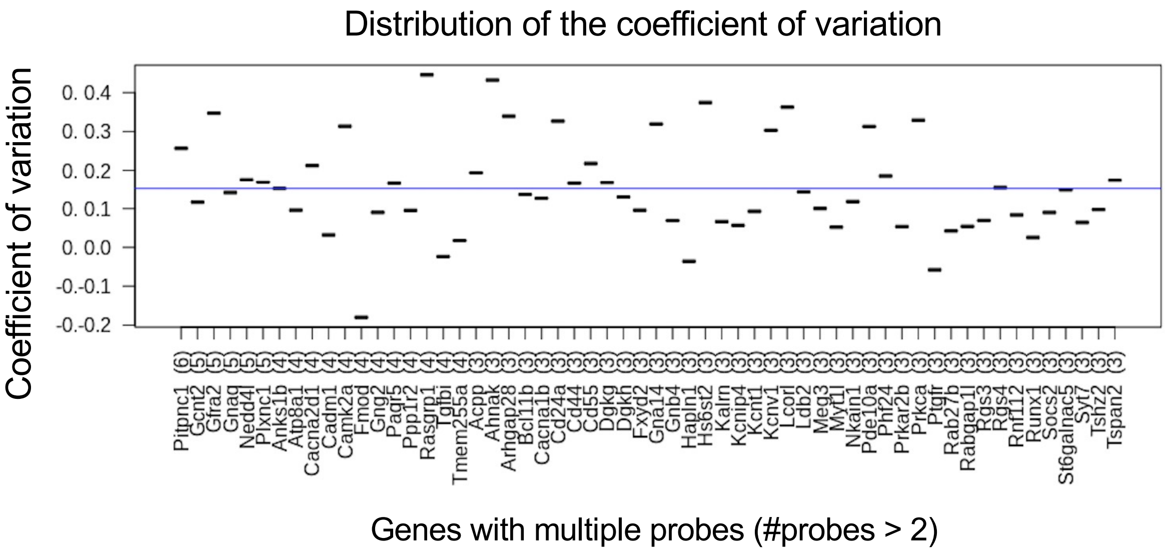

Supplementary Figure 2. Low variability between probes representing the same gene. Distribution of the coefficient of variation (CV) for genes with multiple probes values. CVs are calculated using significance ($p < 0.05$). Genes with more than 2 probes are shown in this figure. Numbers near the gene symbols show how many probes represented each gene. Blue line shows the average CV.
