# Supplementary material for: Transcriptomic and proteomic profiling of NaV1.8-expressing mouse nociceptors
Source: Front Mol Neurosci. 2022 Oct 11;15:1002842. doi: 10.3389/fnmol.2022.1002842 (PMC9593034; doi:10.3389/fnmol.2022.1002842)
Supplement: Supplementary file 14 [file Image_3.pdf]

Supplementary Figure 3

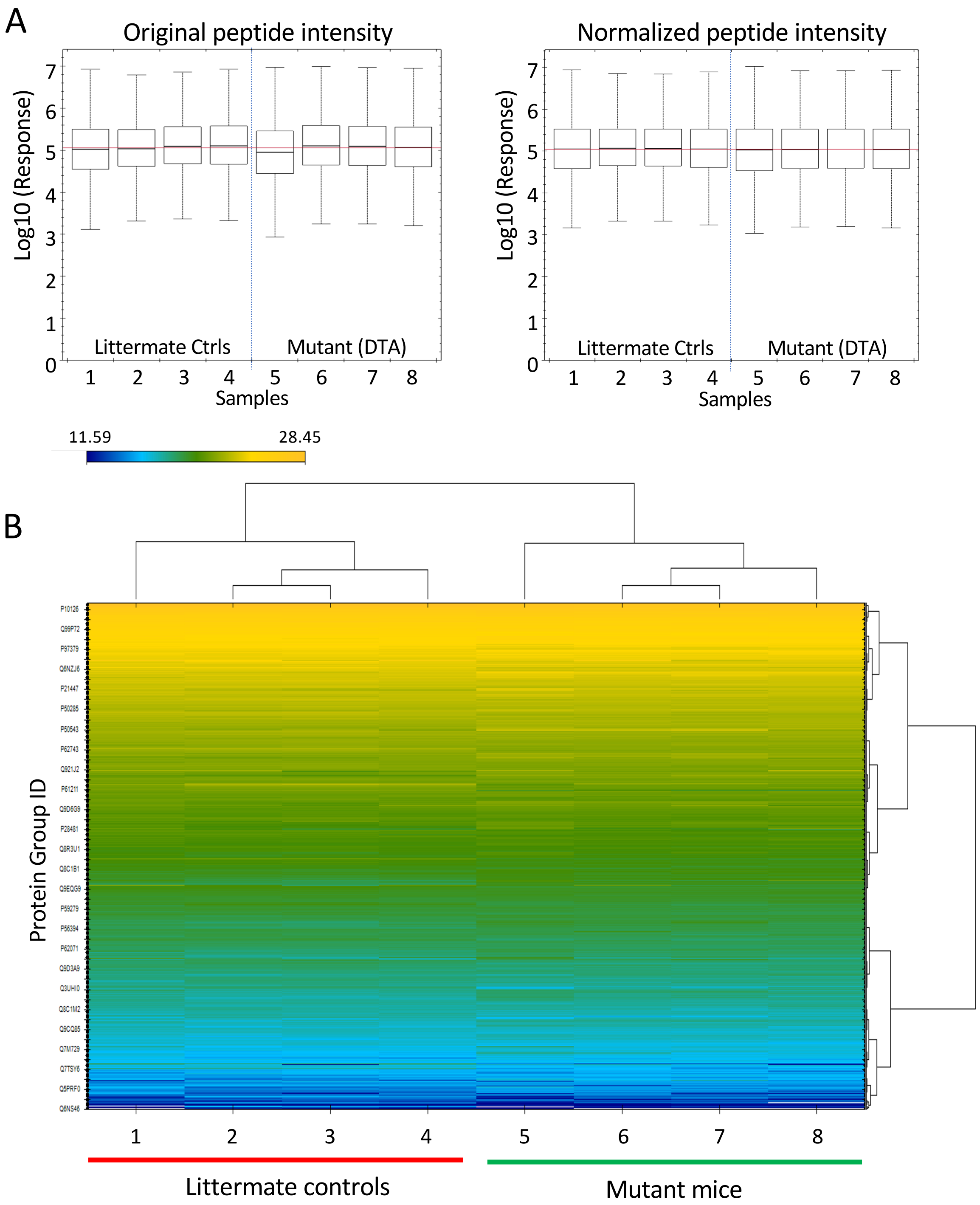

Supplementary Figure 3. (A) Peptide Intensity. Data quality was analyzed using directDIA analysis in Spectronaut X with default setting and local regression normalization (Callister et al., 2006 DOI: 10.1021/pr050300l). Normalization of the peptide intensity was performed to compensate for loading and instrument performance fluctuations. A comparison of peptide intensities (log10) of original data (left panel) and normalized data (right panel) demonstrates that acquisition stability was good and minor normalization was carried out. **(B)** Heat map and unsupervised clustering of each sample on the level of protein group data. Note the separation between the littermate control (Ctrl) and mutant samples. The heatmap was generated using heatmap.2 of the ggplot2 package (https://ggplot2.tidyverse.org) in the statistical package R. Distance was calculated using the “manhattan” method, the clustering using “ward.D”
